# Supplementary material for: An adaptive sliding mode controller with free-will arbitrary time convergence for three-phase rectifiers in autonomous agricultural vehicles
Source: PLoS One. 2025 Aug 22;20(8):e0330424. doi: 10.1371/journal.pone.0330424 (PMC12373180; doi:10.1371/journal.pone.0330424)
Supplement: Attachment — Submitted filename: Response to Reviewers.pdf [file pone.0330424.s002.pdf]

Response to reviewer 1

Item no.	Reviewer Comment	Action
1	The Abstract is quite elaborate. Abstract must be the summary of the entire paper in a concise and compact form. Thus, it is suggested to make the abstract compact by mainly focusing on the novelty of the proposed work.	Thank you for the valuable feedback. We have revised the Abstract to make it more concise, focusing on the main objective, the novelty of the proposed controller, and key results to enhance clarity and impact. (in the abstract section).
2	The Introduction is good, and the literature reviews are well highlighted. However, the entire Introduction is written in a single paragraph. It is suggested to break it in small paragraphs. In the last paragraph, the contribution of the proposed work along with the organization of the rest parts the paper is to be included.	Thank you for your helpful suggestion. We have revised the Introduction by splitting it into smaller paragraphs and added a final paragraph highlighting the main contributions and structure of the paper, as recommended. (From line 1 to line 114).
3	In Figure 1, the images just above the blocks "Three Phase Rectifier" and "BMS" are absolutely blurred and nothing is visible. It is suggested to include HD images for those two pictures.	Thank you for pointing this out. We have replaced the two blurred images with high-definition (HD) versions to ensure all details are visible. (From line 127 to line 128).
4	In the sentence on page no. 3: "The phase rectifier can be represented in the below three equations [28]:", there are four equations (1-4) not three. Correct.	We have corrected the sentence to accurately state that four equations, not three, represent the rectifier. (line 132)
5	Below equation (13), replace r and e by r and e.	Done with thanks.(From line 176 to line 177)
6	Below Equation (13), there is a long text. Break in paragraphs.	We have revised the long text below Equation (13) and separated it into three clearer and logically structured paragraphs to improve readability and clarity. (From line 175 to line 204)
7	Below equation 31, the sentence: "The advantages of using such an addition are It will provide a free-will convergence to the error of the DC-link voltage of the rectifier, which is the desired state (Vdc →V∗dc) within the arbitrary time (Tfadjusted) regardless of initial conditions or disturbances." is ambiguous and not clear. Rewrite it. It is suggested to write all advantages either by using bullets or by numbering.	As suggested, we have revised the text below Equation (31) to improve clarity and remove ambiguity. The advantages are now listed using bullet points for better readability and structured presentation. (From line 342 to line 350)
8	Place equation 8 at the center of the page.	Thank you for your suggestion. We want to clarify that we use the PLOS ONE LaTeX template, which automatically centers all equations using the \begin{Equation}...\end{Equation} environment. Equation (8) is now centered on following the journal's formatting standards. We also centered all the equations in the paper. (Line 149)

9	The font size of Eqn. 10 is not same like other equations. Correct Eqn. 10. Also place the equation number 10 at right aligned position.	Thank you for pointing this out. We acknowledge that Equation (10) was initially written in-line using display math syntax, which caused a mismatch in font size and misalignment of the equation number. We have now corrected this by formatting Equation (10) using the standard \begin{Equation}...\end{Equation} environment to ensure consistency in font size and alignment with other equations following PLOS ONE formatting guidelines. (Line 166)
10	Below Fig. 6, the authors have written "Figure 6 illustrates the iq− iq harmonic current detection algorithm based on instantaneous reactive power theory." However, the figure caption describes the block diagram. It is ambiguous.	It has been corrected as follows: (In Fig 6, the reference voltage of the controlled rectifier is varied according to the control signals generated by the designed Battery Management System (BMS). For example, if the State of Charge (SoC) of the 24-volt battery falls below the threshold defined by the BMS algorithm, the system adjusts the reference voltage of the AFWATSMC algorithm to 28 volts. Similarly, if the SoC of the 12-volt battery is low, the BMS sends a control signal to change the reference voltage to 15 volts. Simultaneously, the load condition of each battery is monitored to verify whether it meets the predefined loading criteria. This figure demonstrates the robustness of the proposed algorithm against various types of disturbances like reference voltage variation and load fluctuation.). (From line 600 to line 611)
11	Fig 3 is blurred. Replace it by HD image.	Thank you for pointing this out. We have replaced Fig 3 with high-definition (HD) versions to ensure all details are visible. (From line 473 to line 474)
12	In step c:Error Threshold check of the algorithm for the AFWATSMC for the sentence: "Next, the algorithm evaluate whether the error (edc) exceeds the predefined threshold set bt the algorithm" what "bt" means.	Thank you for pointing out this oversight. "bt" is a typographical error, and the correct word should be "by." We appreciate your attention to detail. (Line 453)
13	On page 15 for the sentence: "Finally, the same 15 v as a reference voltage and loading conditions were applied to both systems." 15 v will be replaced by 15 V.	Done with thanks. (Line 561)
14	Explain Fig. 5 and Fig. 6 in more detail.	Done, with thanks, for Figure 5 (In this figure, the speed is varied according to the measured speed drive cycle of the grabber. Using the *Signal Builder* block in MATLAB Simulink, an emulation of this speed drive cycle is achieved. Although the speed fluctuates, the output DC voltage remains constant at 20 volts. This result demonstrates the robustness of the proposed AFWATSMC.) , and for Figure 6 (In Fig 6, the reference voltage of the controlled rectifier is varied according to the control signals generated by the designed Battery Management System (BMS). For example, if the State

		of Charge (SoC) of the 24-volt battery falls below the threshold defined by the BMS algorithm, the system adjusts the reference voltage of the AFWATSMC algorithm to 28 volts. Similarly, if the SoC of the 12-volt battery is low, the BMS sends a control signal to change the reference voltage to 15 volts. Simultaneously, the load condition of each battery is monitored to verify whether it meets the predefined loading criteria. This figure demonstrates the robustness of the proposed algorithm against various types of disturbances like reference voltage variation and load fluctuation.). (From line 592 to line 611)
15	Replace all figures of Fig. 7 with HD images and large font for axes indentations.	We thank the reviewer for the helpful suggestion. Fig. 7 has been updated with high-resolution images and enlarged axis fonts for better clarity. Additionally, we have reformatted the figure according to PLOS ONE guideline by centering subscriptions and placing the bold main caption below the figure. These improvements address the comment and enhance the figure's readability and presentation. (From line 625 to line 626)
16	Write all parameters in Table 2 in italics font.	Thank you for pointing this out. We have corrected the formatting of all parameter symbols in Table 4. The parameters k_1, k_2, η_1, R, L, and C are now presented in italic font following scientific writing conventions. (From line 625 to line 626) (Note table 2 is now table 4 because we add 2 other tables)
17	Change the yellow color of all plots by some other deep colors like red or black. Also increase the depth of colors to understand the comparison plots between AFWATSMC and ASMC.	Thank you for your valuable suggestion. In response, we have changed all yellow-colored plots to deeper and more distinguishable colors, such as red and black, to improve visual clarity. Additionally, we have enhanced the line thickness and contrast to ensure better visibility of the comparison plots between AFWATSMC and ASMC. These modifications have been implemented across all relevant figures for better interpretability. (line 598 , line 637 , line 650 , line 664, line 664 , line 671 , line 683 , line 684 , line 690 , line 697 , line 697 , line 710 , line 713).
18	Fig. 9 is absolutely blurred. It can't be accepted for the paper. All left hand side texts are blurred. Replace Fig. 9 by HD images. It is suggested to separate the images and place individually one after another with proper figure number.	Thank you for your valuable feedback. In response to your comment regarding **Figure 9**, we have carefully revised it by splitting the original figure into two high-resolution images (**now Fig 9_a and Fig 9_b**) to improve clarity and readability.<br>We hope this revision resolves the concern and enhances the overall quality and presentation of the manuscript.(From line 643 to line 644)

19	Write concluding remarks based on Table 4.	Thank you for your insightful suggestion. In response, we added a concluding paragraph summarizing the key findings based on Table 4. The comparative analysis demonstrates that the proposed AFWATSMC outperforms the ASMC in all evaluated criteria, including faster transient response, lower overshoot, shorter settling time, and improved peak performance. These remarks are now included in the revised manuscript to emphasize the effectiveness and superiority of the proposed control method. (From line 803 to line 805)
20	Include a comparative table with some recently published works based on some common parameters to justify the novelty of the proposed work.	We sincerely thank the reviewer for this valuable observation. We have expanded the literature review section to include a comprehensive comparative table summarizing recent studies involving sliding mode controllers (SMC) applied to three-phase rectifiers, including applications such as model predictive control (MPC), hybrid control strategies, and grid-connected systems. This is clear in Table 7. (From line 717 to line 734).

Response to Reviewer 2

Item no.	Reviewer Comment	Action
1	Structure and Flow:<br>• The manuscript is dense and lengthy, with long paragraphs and limited use of subheadings, making it difficult to navigate. Sections such as the Introduction and methods should be broken down into clearly numbered subsections to improve organization and flow.<br><br>The title is confusing and overly complex. Consider rephrasing to emphasize the application and novelty (e.g., "An Adaptive Sliding Mode Controller with Arbitrary-	We sincerely appreciate the reviewer's valuable observation regarding the manuscript's organization and structure. In response, we have shortened and refined the Abstract and Introduction to improve clarity and focus. Additionally, long paragraphs have been split into smaller, more manageable ones to enhance readability and flow. Regarding the suggestion to number sections and subsections, we respectfully note that we followed the official PLOS ONE formatting guidelines, which do not include numbered headings. (Lines 2, 10,25,38.48,61,68,78,96 in the introductions and lines 176,185,196,216,220,240,319,326,342,362,372,430,530,539, in the methodology)<br><br>Thank you for your valuable suggestion. We agree that the original title was somewhat lengthy and could benefit from improved clarity and emphasis

	Time Convergence for Three-Phase Rectifiers in Autonomous Agricultural Vehicles").	on the core contribution of the study. Based on your recommendation, we have revised the title to:<br><br>**"An Adaptive Sliding Mode Controller with free-will Arbitrary Time Convergence for Three-Phase Rectifiers in Autonomous Agricultural Vehicles"**<br><br>We add the free-will term as explained in our response to your comment no. 5.
2	Introduction:<br>• The Introduction sets a solid context about the challenges in oil palm harvesting and the use of MBGs, but it lacks citations of recent work in control systems for autonomous agricultural machinery or rectifier control.<br><br>• While the motivation is clear, the novelty of the proposed controller should be stated explicitly. What differentiates this controller from other SMC variants or existing rectifier control strategies?	We thank the reviewer for the insightful comment. In response, we have expanded the Introduction by incorporating a dedication to autonomous agricultural machinery and rectifiers. For autonomous agrarian machinery, we added references 12,13 and 14 (from line 24 to line 46). For the rectifier, we added three references (ref 32,33,34) in the Introduction and Table 7 to compare the results of the previous work related to SMC-based control of three-phase rectifiers with the proposed new AFWATSMC.<br>These additions help contextualize our contribution within the broader scope of current research in control systems for autonomous applications.<br><br>We thank the reviewer for this valuable comment. In response, we have explicitly stated the novelty and unique contributions of the proposed AFWATSMC algorithm in Table 7(From line 719 to line 734). Specifically, we emphasize that this is the first known application of the Adaptive Free-Will Arbitrary Time Sliding Mode Control (AFWATSMC) in power electronic systems, particularly for PWM-based three-phase rectifiers. Unlike existing SMC variants, our proposed controller enables user-defined convergence time independent of system parameters, disturbances, or initial conditions. Additionally, we highlight our novel integration of GA and PSO for tuning the AFWATSMC parameters, enhancing its robustness and practical utility in nonlinear, disturbance-prone environments. These clarifications now make the innovation and contribution of our work more transparent and distinguishable from existing strategies. We added these contributions in the Abstract, end of the introduction (from 99 line to line 109), and conclusion (from line 808 to line 812)

3	• The key innovation lies in integrating free-will arbitrary convergence time with an adaptive SMC structure. However, this should be highlighted more clearly in the abstract and conclusion.	We appreciate the reviewer's insightful comment. We have explicitly emphasized the key innovation in the Abstract and conclusion to address this. Specifically, we clarified that the core novelty lies in combining the free-will arbitrary convergence time feature with an adaptive sliding mode controller structure, allowing the user to define convergence time independently of system disturbances and initial conditions. We added these contributions in the Abstract, end of the introduction (from 99 line to line 109), and conclusion (from line 808 to line 812)
	• The mathematical derivations are comprehensive, but the novelty gets buried in formula-heavy sections. A concise summary (e.g., bullet points or a table) comparing your approach to ASMC or other SMC methods would enhance clarity.	We appreciate your valuable suggestion. In response, we have added **Table 1** to the revised manuscript as a clear and concise summary comparing the **mathematical representation of the proposed AFWATSMC** with the conventional **ASMC**. This table highlights key differences in control laws, adaptive terms, convergence strategies, and Lyapunov validation. We believe this addition enhances the clarity and accessibility of the proposed controller's novelty, especially in contrast to traditional sliding mode control approaches. (From line 570 to line 571)
4	• Many figures are not discussed adequately in the text. For example, Figures 5–16 are introduced with minimal analysis or interpretation.	Thank you for your valuable observation. In response to your comment regarding the limited discussion of Figures 5–16, we have thoroughly revised the **Results and Discussion** section to ensure each figure is analyzed and interpreted in the context of the study's objectives. (For Fig 5 and Fig 6 from line 595 to line 611, for Fig 7 from line 616 to line 621, for Fig 8, from line 632 to line 637, for Fig 9, from line 640 to line 643, for Fig 10 from line 645 to line 650, for fig 11, and fig 12, from line 652 to line 664, for fig 13 from line 666 to line 671, for fig 14 and 15, from line 673 to line 683 and finally, Fig 16 from line 685 to line 690.)
	• Captions are often too brief or generic. For example,	Thank you for your valuable observation. We have carefully revised the figure captions throughout

	"DC Output Voltage Behavior" doesn't explain the scenario or what distinguishes the behavior shown.<br><br>• Table 1 on convergence lacks context—what does "yes" or "no" convergence mean practically?<br><br>• Consider adding a summary table of all tested conditions and system responses for clarity.	the manuscript to provide more detailed and informative descriptions.<br><br>Thank you for your valuable observation regarding Table 1. We have clarified the context of the "Yes" and "No" entries. These responses indicate whether the system states (DC voltage error) converge to the desired values within the user-defined arbitrary convergence time using the FWAT algorithm.<br>Specifically, "Yes" denotes successful convergence, verified through MATLAB Simulink simulations. We employed a dynamic filtering approach in these tests to monitor and assess convergence behavior under various parameters and initial condition variations. "No" indicates that the system failed to converge to the target state within the specified time due to excessive disturbances or initial deviations.<br><br>Thank you for the insightful suggestion. As recommended, we have included a **summary table** (now added as **Table 2**) that consolidates **all tested conditions** (such as parameter variation, reference voltage changes, load fluctuations, and disturbances) and the corresponding **system responses** under both AFWATSMC and ASMC controllers. This table presents key performance indicators such as **convergence time, total harmonic distortion (THD), ripple factor, overshoot**, and **tracking error**, helping readers quickly compare the effectiveness of the proposed controller under different scenarios. (From line 594 to line 595)
5	English Language and Style:<br>• The manuscript suffers from grammatical inconsistencies, awkward phrasing, and run-on sentences (e.g., the sentence starting with "The advantages of using such an addition are...").<br>• Please consider professional English language editing. Use consistent past tense for experimental descriptions and present tense for general truths.	Thank you for your insightful comments regarding the language and style of the manuscript. The entire manuscript has been professionally proofread and edited by a certified English language editing service. All grammatical inconsistencies, awkward phrasing, and tense issues have been carefully corrected. We have ensured consistent use of past tense for experimental descriptions and present tense for general statements. Upon request, a certificate from the proofreading service is available to confirm that a qualified language editing company completed these revisions.

<table>
<tr>
<td></td>
<td>• Terms like "free-will arbitrary time" are unconventional in engineering literature. You may want to explain this term clearly or use more standard terminology such as "user-defined convergence time."</td>
<td>
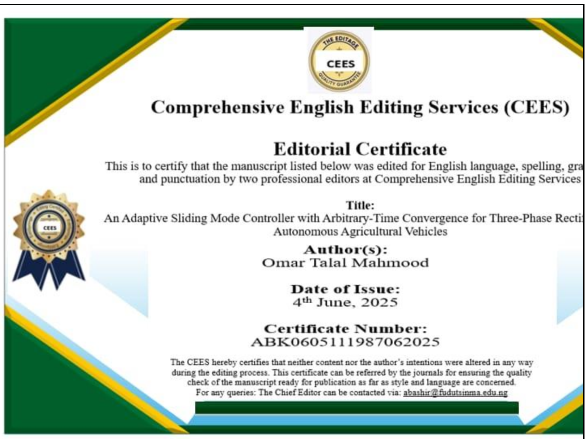

Comprehensive English Editing Services (CEES)

Editorial Certificate

This is to certify that the manuscript listed below was edited for English language, spelling, gra[illegible] and punctuation by two professional editors at Comprehensive English Editing Services

Title:
An Adaptive Sliding Mode Controller with Arbitrary-Time Convergence for Three-Phase Recti[illegible] Autonomous Agricultural Vehicles

Author(s):
Omar Talal Mahmood

Date of Issue:
4th June, 2025

Certificate Number:
ABK0605111987062025

The CEES hereby certifies that neither content nor the author's intentions were altered in any way during the editing process. This certificate can be referred by the journals for ensuring the quality check of the manuscript ready for publication as far as style and language are concerned.
For any queries: The Chief Editor can be contacted via: abashir@fudutsinma.edu.ng

We appreciate the reviewer's insightful comment regarding "free-will arbitrary time." While we understand that this phrase may appear unconventional in some areas of engineering literature, it is a term that has gained recognition in recent control systems research. In particular, several recent and high-quality publications have used this terminology to describe control algorithms that achieve convergence in a time frame defined freely and arbitrarily by the designer.

To support our usage, we reference the following works where this terminology is consistently adopted:

1. A. K. Pal et al., "Free-Will Arbitrary Time Terminal Sliding Mode Control," *IEEE Trans. Circuits Syst. II*, vol. 69, no. 7, pp. 3189–3193, 2022.
2. H. Wang et al., "Free-Will Arbitrary Time Adaptive Control for Uncertain Lorenz System," *IEEE Trans. Circuits Syst. II*, vol. 71, no. 1, pp. 191–195, 2024.
3. Y. Feng et al., "Free-will arbitrary time consensus of heterogeneous nonlinear multi-agent systems," *Commun. Nonlinear Sci. Numer. Simul.*, vol. 128, 2024.
4. E. Aslmostafa, M. J. Mirzaei, M. Asadollahi, and M. A. Badamchizadeh, "Stabilization Problem for a Class of Nonlinear MIMO Systems Based on Prescribed-Time Sliding Mode Control," Arabian Journal for Science and Engineering, vol. 47, no. 11, pp. 15083–
</td>
</tr>
</table>

		15094, Jun. 2022, doi: 10.1007/s13369-022-06974-4.<br><br>These references demonstrate that the term "free-will arbitrary time" has become a recognized concept in the control systems domain, particularly in the context of sliding mode control, consensus, and adaptive control algorithms.<br><br>Nonetheless, we understand the reviewer's concern and have clarified this terminology in the revised manuscript to improve clarity for a broader audience. Should the editorial board prefer a more standard term, such as "user-defined convergence time," we remain open to adopting it.
6	Validation and Discussion:<br>• While the controller is validated in simulation and parameter tuning is supported by GA and PSO, the physical implementation is not discussed. Is this purely simulated? If so, limitations should be explicitly stated.<br><br>• The paper would benefit from a quantitative comparison of key performance metrics (e.g., THD, convergence time, ripple factor) between AFWATSMC and conventional controllers.	• Yes, this work is purely simulated using MATLAB Simulink and coding designed to implement the newly proposed AFWATSMC. We added this paragraph to the limitation section of the paper. (Yet, the validation of the AFWATSMC using Matlab programming and Simulink is limited. The experimental setting up required a physical testing environment, hardware, and software components, such as the controlled three-phase rectifier, alternator, robust tools for data collection, and a bright, efficient controller device; this process was not only time-consuming only but also required specific equipment with high-speed switching frequency to reduce the chattering, resource-intensive and needs extended testing time to stand for a wide range of disturbances and system uncertainties. Furthermore, Matlab simulation provides a highly repeatable and controlled testing setting, permitting accurate exploitation of system parameters and disturbances in such a way that it is difficult to achieve experimentally.) , with thanks.<br><br>• we compare the results obtained by the simulation of the new algorithm with the results of another adaptive SMC. The papers that are used in this comparison are :<br>- D. Sakri, H. Laib, S. E. Farhi, and N. Golea, "Sliding mode approach for control and observation of a three-phase AC-DC pulse-width modulation rectifier," Electrical Engineering and Electromechanics, vol. 2023, no. 2, pp. 49–56, 2023, doi: 10.20998/2074-272X.2023.2.08.

		- S. Gadgune and P. M. Joshi, "Control of Three-Phase PWM Rectifier as Virtual Synchronous Machine Using an Integral Sliding Mode Controller," Journal of The Institution of Engineers (India): Series B, vol. 102, no. 2, pp. 193–201, Apr. 2021, doi: 10.1007/s40031-020-00523-z.<br>- A. Ahmane, D. Sakri, and N. Golea, "Direct Power Control based on Super Twisting Sliding Mode Control for Three Phase PWM Rectifier," in 2022 2nd International Conference on Advanced Electrical Engineering, ICAEE 2022, Institute of Electrical and Electronics Engineers Inc., 2022, pp. 1–6. doi: 10.1109/ICAEE53772.2022.9962104.<br>- S. Barkat, A. Tlemçani, and H. Nouri, "Direct power control of the PWM rectifier using sliding mode control," Int. J. Power and Energy Conversion, vol. 2, no. 4, pp. 289–306, 2011, doi: 10.1504/ijpec.2011.041883.<br>We simulated and designed these papers in MATLAB Simulink and extracted the results regarding THD, convergence time, ripple factor, and other parameters, as shown in the result section.<br>This comparison is made regarding the shape and THD content of the AC output voltage and current of the alternator. Furthermore, the DC output response is another parameter to compare with its ripple factor. In addition, the time convergence of the system to zero is also compared. The value of the power factor is also compared using the two algorithms. Moreover, the step response parameters are also used as comparison parameters between the two algorithms. Finally, the effect of the THD on the alternator temperature is also compared using the proposed algorithm with the ASMC.<br>The same control law and PI sliding surface have been implemented in the proposed algorithm. Without adding the (u) term to the AFWATSMC algorithm. The same DPSMC is used in the current Loop and optimized SMC parameters are used for both systems. The same AC and DC filter Parameters have also been used for both systems.
	• More insights should be provided into practical implementation issues: real-time feasibility, computational cost, and integration with embedded systems.	•We thank the reviewer for highlighting the importance of practical implementation aspects. We have extended the limitations section to explicitly discuss the real-time feasibility, computational cost, and challenges in integrating

		the proposed AFWATSMC into embedded systems. These clarifications improve the transparency of the manuscript and provide a realistic scope for future experimental and embedded implementation. (From line 813 to line 845)
7	References:<br>• The literature review is broad but misses key studies on modern SMC applications in rectifiers, such as those using model predictive control, hybrid controllers, or grid-connected converters.<br><br>The reference formatting is inconsistent; ensure all references follow PLOS ONE guidelines.	• We sincerely thank the reviewer for this valuable observation. In response, we have expanded Table 7 to include a comprehensive comparative table summarizing recent studies involving sliding mode controllers (SMC) applied to three-phase rectifiers, including applications such as model predictive control (MPC), hybrid control strategies, and grid-connected systems.<br>This table highlights the key algorithms, control objectives, converter types, and comparative insights, thereby strengthening the contextual background and justifying the novelty of our proposed AFWATSMC approach.<br>We hope this enhancement addresses the reviewer's concern and improves the technical relevance and completeness of the manuscript. (From line to line 717 to line 734)<br><br>• Thank you for pointing out the inconsistency in reference formatting. We have carefully revised all references in the manuscript to fully comply with the **updated** PLOS ONE citation guidelines, which align with the recommendations of the International Committee of Medical Journal Editors (ICMJE), as outlined by the U.S. National Library of Medicine [https://www.nlm.nih.gov/bsd/uniform_requirements.html]. All references consistently follow this standard, including correct formatting of author names, journal titles, volume and issue numbers, page ranges, and DOI links.
8	• Here are five relevant references that the authors might cite to strengthen the novelty and relevance of the harmonic mitigation and control methodology (especially for high-performance rectifier systems). These references would enhance the relevance of your harmonic mitigation discussion and modernize your bibliography:<br>1) Al-Barashi, M., Zou, A., Wang, Y., Luo, W., Shao, N., Tang, Z., & Lu, B. (2025). Magnetic Integrated Multi-Trap Filters Using Mutual Inductance to Mitigate Current Harmonics in Grid-Connected Power Electronics Converters. Energies, 18(2), 423. https://doi.org/10.3390/en18020423.<br>2) Al-Barashi M, Wang Y, Bhutta MS (2024) High-	We thank the reviewer for the valuable recommendations and for directing us to recent high-quality publications relevant to harmonic mitigation in power electronic systems. After carefully reviewing all five references, we have included the following three papers in our revised manuscript, as they directly relate to our focus on harmonic suppression and filter design in controlled rectifier systems:<br><br>1. Al-Barashi M, Wang Y, Bhutta MS (2024) *High-frequency harmonics suppression in high-speed railway*

	frequency harmonics suppression in high-speed railway through magnetic integrated LLCL filter. PLOS ONE 19(6): e0304464. https://doi.org/10.1371/journal.pone.0304464.<br>3) Al-Barashi, M., Wang, Y., Lan, B. et al. Magnetic integrated double-trap filter utilizing the mutual inductance for reducing current harmonics in high-speed railway traction inverters. Sci Rep 14, 10058 (2024). https://doi.org/10.1038/s41598-024-60877-y.<br>4) Al-Barashi, M., Meng, X., Liu, Z., Saeed, M.S.R., Tasiu, I.A., Wu, S.: Enhancing power quality of high-speed railway traction converters by fully integrated T-LCL filter. IET Power Electron. 16, 699–714 (2023). https://doi.org/10.1049/pel2.12415.<br>5) Al-Barashi, M., Wu, S., Liu, Z., Meng, X., Tasiu, I.A.: Magnetic integrated LLCL filter with resonant frequency above Nyquist frequency. IET Power Electron. 15, 1409–1428 (2022). https://doi.org/10.1049/pel2.12313.	*through magnetic integrated LLCL filter*. PLOS ONE 19(6): e0304464.<br>2. Al-Barashi, M., Meng, X., Liu, Z., Saeed, M.S.R., Tasiu, I.A., Wu, S. (2023) *Enhancing power quality of high-speed railway traction converters by fully integrated T-LCL filter*. IET Power Electronics, 16, 699–714.<br>3. Al-Barashi, M., Wu, S., Liu, Z., Meng, X., Tasiu, I.A. (2022) *Magnetic integrated LLCL filter with resonant frequency above Nyquist frequency*. IET Power Electronics, 15, 1409–1428.<br><br>These papers provided valuable insights into modern filter design methodologies and motivated improvements in our harmonic suppression strategy. We have cited and discussed them (as ref 32 ,33,34) in the context of advanced filtering solutions for rectifier-based systems.<br><br>Regarding the other two references:<br><br>• Al-Barashi et al. (2025) *"Magnetic Integrated Multi-Trap Filters..."* and<br>• Al-Barashi et al. (2024) *"Magnetic integrated double-trap filter..."*<br><br>While these works are technically rigorous and innovative, they focus primarily on general grid-connected converter topologies and traction inverters with multi-trap configurations. However, they do not directly align with our paper's specific scope, which targets **harmonic mitigation within controlled rectifier systems** using sliding mode control strategies. Therefore, we respectfully chose not to include these two references to maintain coherence and relevance.<br><br>We hope this selection and justification meet the reviewer's expectations and strengthen our revised manuscript's clarity and technical depth. (line 91)
