# Supplementary material for: An adaptive sliding mode controller with free-will arbitrary time convergence for three-phase rectifiers in autonomous agricultural vehicles
Source: PLoS One. 2025 Aug 22;20(8):e0330424. doi: 10.1371/journal.pone.0330424 (PMC12373180; doi:10.1371/journal.pone.0330424)
Supplement: Attachment — Submitted filename: Response_to_Reviewers_auresp_2.pdf [file pone.0330424.s003.pdf]

Response to reviewer 1

Item no.	Reviewer Comment	Action
1	It is suggested to include one more row at the end of Table 7 mentioning "This Work" and then write the supporting results in subsequent columns.	Done with thanks as in page 31 from line 733 to line 734.

Response to Reviewer 2

Item no.	Reviewer Comment	Action
1	In technical writing, it is not true to write “we, I, our, etc.” There are still some places that were not corrected, or some places in the new content were not written considering this comment.	Corrected with thanks as below:<br><br>- For (we), it has been corrected as in lines (285,315,423,425).<br>- For (our) it has been corrected as in line (106,215,360,371,728).<br>- For (I) it has been fixed as in line (850)
