# Supplementary material for: An adaptive sliding mode controller with free-will arbitrary time convergence for three-phase rectifiers in autonomous agricultural vehicles
Source: PLoS One. 2025 Aug 22;20(8):e0330424. doi: 10.1371/journal.pone.0330424 (PMC12373180; doi:10.1371/journal.pone.0330424)
Supplement: Attachment — Submitted filename: PONE-D-25-20009_reviewer Comments.docx [file pone.0330424.s001.docx]

This manuscript proposes a novel **Adaptive Free-Will Arbitrary Time Sliding Mode Controller (AFWATSMC)** for a three-phase rectifier used in an oil palm autonomous grabber. The aim is to improve DC voltage regulation and reduce total harmonic distortion (THD) while addressing thermal issues due to excessive loading on the alternator in a robotic mobile platform. The paper includes an original control scheme with adaptive terms and modified convergence logic using MATLAB/Simulink modeling, genetic algorithms (GA), and particle swarm optimization (PSO) for parameter tuning. While the topic is timely and interesting, and the integration of FWAT with SMC is original, the manuscript **requires significant revisions** to improve readability, clarity, and alignment with standard engineering practices.

**Major Comments**

1. **Structure and Flow:**
   - The manuscript is **dense and lengthy**, with long paragraphs and limited use of subheadings, making it difficult to navigate. Sections such as the introduction and methods should be broken down into **clearly numbered subsections** to improve organization and flow.
   - The **title is confusing and overly complex**. Consider rephrasing to emphasize the application and novelty (e.g., *"An Adaptive Sliding Mode Controller with Arbitrary-Time Convergence for Three-Phase Rectifiers in Autonomous Agricultural Vehicles"*).
2. **Introduction:**
   - The introduction sets a solid context about the challenges in oil palm harvesting and the use of MBGs, but it **lacks citations** of recent work in control systems for autonomous agricultural machinery or rectifier control.
   - While the motivation is clear, the **novelty of the proposed controller should be stated explicitly**. What differentiates this controller from other SMC variants or existing rectifier control strategies?
3. **Novelty and Contribution:**
   - The key innovation lies in integrating **free-will arbitrary convergence time** with an adaptive SMC structure. However, this should be **highlighted more clearly in the abstract and conclusion**.
   - The **mathematical derivations are comprehensive**, but the novelty gets buried in formula-heavy sections. A concise summary (e.g., bullet points or a table) comparing your approach to ASMC or other SMC methods would enhance clarity.
4. **Figures and Tables:**
   - Many figures are **not discussed adequately in the text**. For example, Figures 5–16 are introduced with minimal analysis or interpretation.
   - Captions are often **too brief or generic**. For example, “DC Output Voltage Behavior” doesn’t explain the scenario or what distinguishes the behavior shown.
   - **Table 1** on convergence lacks context—what does “yes” or “no” convergence mean practically?
   - Consider adding **a summary table** of all tested conditions and system responses for clarity.
5. **English Language and Style:**
   - The manuscript suffers from **grammatical inconsistencies**, awkward phrasing, and run-on sentences (e.g., the sentence starting with "The advantages of using such an addition are...").
   - Please consider professional English language editing. Use **consistent past tense** for experimental descriptions and present tense for general truths.
   - Terms like "free-will arbitrary time" are unconventional in engineering literature. You may want to explain this term clearly or use more standard terminology such as “user-defined convergence time.”
6. **Validation and Discussion:**
   - While the controller is validated in simulation and parameter tuning is supported by GA and PSO, the **physical implementation is not discussed**. Is this purely simulated? If so, limitations should be explicitly stated.
   - The paper would benefit from a **quantitative comparison** of key performance metrics (e.g., THD, convergence time, ripple factor) between AFWATSMC and conventional controllers.
   - More insights should be provided into **practical implementation issues**: real-time feasibility, computational cost, and integration with embedded systems.
7. **References:**
   - The literature review is broad but **misses key studies on modern SMC applications in rectifiers**, such as those using model predictive control, hybrid controllers, or grid-connected converters.
   - The reference formatting is inconsistent; ensure all references follow PLOS ONE guidelines.
8. **Suggested References (for Authors to Consider Including):**
   - Here are five relevant references that the authors might cite to strengthen the novelty and relevance of the harmonic mitigation and control methodology (especially for high-performance rectifier systems). These references would enhance the relevance of your harmonic mitigation discussion and modernize your bibliography:
1) Al-Barashi, M., Zou, A., Wang, Y., Luo, W., Shao, N., Tang, Z., & Lu, B. (2025). Magnetic Integrated Multi-Trap Filters Using Mutual Inductance to Mitigate Current Harmonics in Grid-Connected Power Electronics Converters. *Energies*, *18*(2), 423. <https://doi.org/10.3390/en18020423>.
2) Al-Barashi M, Wang Y, Bhutta MS (2024) High-frequency harmonics suppression in high-speed railway through magnetic integrated *LLCL* filter. PLOS ONE 19(6): e0304464. <https://doi.org/10.1371/journal.pone.0304464>.
3) Al-Barashi, M., Wang, Y., Lan, B. *et al.* Magnetic integrated double-trap filter utilizing the mutual inductance for reducing current harmonics in high-speed railway traction inverters. *Sci Rep* 14, 10058 (2024). <https://doi.org/10.1038/s41598-024-60877-y>.
4) Al-Barashi, M., Meng, X., Liu, Z., Saeed, M.S.R., Tasiu, I.A., Wu, S.: Enhancing power quality of high-speed railway traction converters by fully integrated T-LCL filter. IET Power Electron. 16, 699–714 (2023). <https://doi.org/10.1049/pel2.12415>.
5) Al-Barashi, M., Wu, S., Liu, Z., Meng, X., Tasiu, I.A.: Magnetic integrated LLCL filter with resonant frequency above Nyquist frequency. IET Power Electron. 15, 1409–1428 (2022). <https://doi.org/10.1049/pel2.12313>.

**Summary and Recommendation**

- This manuscript presents a potentially impactful control strategy for improving power quality in robotic agricultural platforms. However, in its current form, the manuscript suffers from **lack of clarity, verbosity, and insufficient comparative discussion**.

**Recommendation: Major Revision**

- The paper has potential, but it requires significant reorganization, improved English usage, a stronger literature review, and clearer highlighting of contributions before being suitable for publication.
